# Fermented Quinoa and Canihua in Plant‐Based Diets Increase Iron and Zinc Bioavailability in Growing Rats

**DOI:** 10.1002/fsn3.4514

**Published:** 2024-10-18

**Authors:** Vanesa Castro‐Alba, Mirian Vargas, Ann‐Sofie Sandberg, Daysi Perez‐Rea, Björn Bergenståhl, Yvonne Granfeldt, Claudia E. Lazarte

**Affiliations:** ^1^ Division Food and Pharma, Department of Process and Life Science Engineering, Faculty of Engineering LTH Lund University Lund Sweden; ^2^ Food and Natural Products Center University of San Simón Cochabamba Bolivia; ^3^ Division of Food and Nutrition Science, Department of Biology and Biological Engineering Chalmers University of Technology Göteborg Sweden

**Keywords:** bioavailability, canihua, fermented pseudocereals, iron, phytate, quinoa, zinc

## Abstract

This study aimed at evaluating the effects of non‐fermented and fermented pseudocereal flours, quinoa and canihua, on iron and zinc bioavailability in Wistar rats. Two diets prepared with 92% fermented quinoa or 79.5% fermented canihua were compared with diets prepared with the same amount of non‐fermented pseudocereals. Other two quinoa diets were prepared with 60% non‐fermented or fermented quinoa and compared with a refence diet which was free of phytates. Body weight, feed efficiency ratio, and the absorption, retention and bioavailability of iron and zinc were evaluated. While body weight and feed efficiency ratio were higher (*p* < 0.05) in animals after non‐fermented diets, the results of mineral absorption and bioavailability were consistently higher in the diets containing fermented pseudocereals. Iron concentration in the livers of animals after the fermented quinoa (92%) and canihua diet (79.5%), were 34% and 30% higher than after the diets with non‐fermented pseudocereals. Zinc bioavailability, indicated by zinc in femur of animals fed the 60% fermented quinoa diet was 53.2 μg g^−1^ Zn_Int_ g^−1^ BW, comparable to that in animals fed a reference diet with no phytates (58.2 μg g^−1^ Zn_Int_ g^−1^ BW), and significantly higher (*p* < 0.05) than in animals fed the non‐fermented quinoa diet (34.5 μg g^−1^ Zn_Int_ g^−1^ BW). Zinc bioavailability was mainly influenced by phytate content in the diet (*R* ^2^ = 0.665 and *p* = 0.000). The retention of iron in the liver (2220 μg g^−1^ Fe_Int_ g^−1^ BW) was higher in the diet containing 60% of fermented quinoa than in the non‐fermented diet (1429 μg g^−1^ Zn_Int_ g^−1^ BW). Differences in iron absorption were mainly impacted by iron content in the diets (*R* ^2^ = 0.828 and *p* = 0.000). In conclusion, the addition of fermented pseudocereals to diets increased the bioavailability of iron and zinc in Wistar rats. These findings will encourage further research into fermented pseudocereals and their potential health effects.

## Graphical abstract

The addition of fermented pseudocereals in diets may increase the bioavailability of iron and zinc in Wistar rats. The main factors affecting iron bioavailability are its content and likely the content of organic acids. Bioavailability of zinc was mainly influenced by the phytate content of the diets

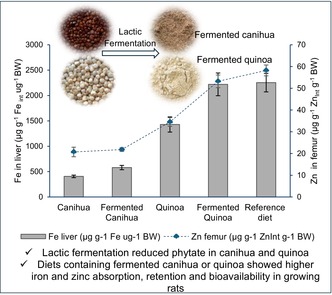

## 1 Introduction

Deficiency of iron and zinc is a common nutritional disorder among women and children in developing countries. Deficiency of these minerals, which are essential micro‐minerals for human growth, development of the immune system and cognitive development, is directly related to dietary intake and content of mineral inhibitors, named phytate, tannins, oxalates and polyphenols (Hurrell and Egli 2010; Lönnerdal 2000). The problem is that although plant‐based diets, containing mainly cereals, legumes, and tubers, have a relatively high content of minerals, these foods also contain inhibitors that reduce the mineral bioavailability in the human body (Chungchunlam and Moughan 2023). The main iron and zinc inhibitor is phytate, a compound that binds to these essential dietary minerals to form insoluble complexes at the physiological conditions in the small intestine and therefore makes them unavailable for absorption (Auer et al. 2024; Sandberg 2002). To improve the bioavailability of iron and zinc, the phytate content of plant‐based foods included in the diet should be reduced. In order to achieve this, dietary processing strategies can be applied to foods before consumption.

Quinoa “*Chenopodium quinoa* Willd.,” and canihua “*Chenopodium pallidicaule*” are pseudocereals of Andean origin, they are gluten‐free crops with relatively high content of good quality proteins, iron and zinc (Castro‐Alba, Lazarte, Bergenståhl, and Granfeldt 2019; Gomez Cahuata, Rosas‐Quina, and Pachari Vera 2022; Schmidt et al. 2023). It has also been reported that both quinoa and canihua grains are major sources of phenolic compounds, that may promote various health benefits (Repo‐Carrasco‐Valencia and Vidaurre‐Ruiz 2021). It is therefore of interest to promote the consumption of these pseudocereals as part of the plant‐based diets of inhabitants of developing countries as well as vegan and vegetarian populations. Further, these nutrient‐rich crops have been recognized for their potential to counteract mineral deficiencies, to achieve dietary diversity and better health (Rahmatov and Lazarte 2023). Although moderate, the phytate content of quinoa and canihua may impair the bioavailability of essential minerals. To markedly improve iron bioavailability the phytate reduction must be virtually complete (Hurrell and Egli 2010). Nowadays, one of the most effective processes to achieve phytate reduction in these pseudocereals is through lactic acid fermentation (Ayub, Castro‐Alba, and Lazarte 2021; Castro‐Alba, Lazarte, Perez‐Rea, Carlsson, et al. 2019; Castro‐Alba, Lazarte, Perez‐Rea, Sandberg, et al. 2019), which procures optimal conditions for the activation of the endogenous plant phytase. This enzyme is present in the raw grains; furthermore, it can be created by microorganisms like lactic acid bacteria during fermentation (Sandberg 2002). The benefit of an activated phytase is that it has the ability to hydrolyze the phytate molecule, leading to less phosphorylated compounds with lower affinity to divalent minerals (Persson et al. 1998), thereby reducing the inhibitory effect of phytate on iron and zinc absorption (McClung et al. 2006; Sandberg et al. 1999).

The influence of lactic acid fermentation on mineral bioaccessibility and bioavailability has been shown for different plant‐based foods (Castro‐Alba, Lazarte, Perez‐Rea, Carlsson, et al. 2019; Lazarte, Castro‐Alba, and Granfeldt 2021; Samtiya et al. 2021; Scheers et al. 2016). Fermented foods, mainly cereals, legumes, and tubers with low phytate content have been mainly studied for their in vitro bioaccessibility of iron and zinc (Castro‐Alba, Lazarte, Perez‐Rea, Carlsson, et al. 2019; Hemalatha, Platel, and Srinivasan 2007), couple of studies reported the in vivo bioavailability in animals (Lazarte, Vargas, and Granfeldt 2015; Tesan et al. 2009), and few in humans (Scheers et al. 2016). However, iron and zinc bioavailability from fermented quinoa and canihua have not been considered in in vivo studies. Regarding the accessibility of minerals in fermented quinoa, some studies have performed quinoa fermentation using different types of lactic acid bacteria (Castro‐Alba, Lazarte, Perez‐Rea, Carlsson, et al. 2019; Valencia et al. 1999), but these studies did not include in vivo trials. Thus, it remains unclear how effective the fermented quinoa and canihua flour are in improving the absorption of iron and zinc in the small intestine. This study, therefore, aims to evaluate the effect of fermentation of quinoa and canihua flour on the bioavailability of iron and zinc using an animal model. The in vivo evaluation was conducted in growing Wistar rats fed non‐fermented or fermented quinoa and canihua diets. The in vivo absorption of iron and zinc was evaluated in two animal studies. Study 1 was carried out to better understand the effect of the type of pseudocereal with high or reduced phytate content on the absorption of iron and zinc. The diets evaluated in study 1 were formulated with pseudocereal flour (non‐fermented or fermented) as the main source of nutrients, particularly proteins and minerals. The diets evaluated in study 2 attempted to emulate a plant‐based diet closer to reality, which also includes dairy foods. In study 2, two quinoa diets with high or reduced phytate content were also compared with a reference diet, which was phytate‐free and mainly composed of milk powder.

## 2 Material and Methods

### 2.1 Food Material and Diets

Fermented quinoa or canihua flours were produced following the procedure described by Castro‐Alba, Lazarte, Perez‐Rea, Carlsson, et al. (2019). For study 1, a suspension of pseudocereal flour and deionized water (ratio 1:2 m V^−1^) was inoculated with *Lactobacillus plantarum* 299v and fermented at 30°C for 24 h for quinoa, and 12 h for canihua. Then, the fermented suspensions were dried at 60°C for 4 h and, milled (500 μm). The acidity, expressed as lactic acid content was determined following the procedure described by Castro‐Alba, Lazarte, Perez‐Rea, Carlsson, et al. (2019), briefly 10% (w/v) suspensions of fermented flours and water were prepared, aliquots of these suspensions were titrated with sodium hydroxide 0.10 N. Lactic acid percent was calculated with the equivalence 1.0 mL of 0.10 N NaOH to 9.0 × 10^−3^ g lactic acid. The lactic acid content and pH were 75.2 g kg^−1^ and 3.82 for fermented quinoa and 80.4 g kg^−1^ and 3.90 for fermented canihua. For study 2, fermented quinoa flour was obtained with the same procedure, with the difference that the fermentation time was reduced to 4 h in order to obtain less acidic/sour fermented flour (40 g kg^−1^ lactic acid) with pH 4.9, this procedure was previously optimized by Castro‐Alba, Lazarte, Perez‐Rea, Sandberg, et al. (2019).

The experimental diets evaluated in the two in vivo studies are shown in Table 1. In study 1, four diets which contained either fermented or non‐fermented quinoa and canihua flours were evaluated. Non‐fermented diets were prepared with dry roasted quinoa (Q‐1) or dry roasted canihua flours (C‐1). Fermented diets were prepared with fermented‐dry roasted quinoa (FQ‐1) or fermented‐dry roasted canihua flours (FC‐1), additional ingredients in the four diets were cane sugar and sunflower oil. In study 2, a shorter fermentation time was applied, as it was shown in our previous research (Castro‐Alba, Lazarte, Perez‐Rea, Sandberg, et al. 2019) that 4‐h fermentation brought better sensory properties than long‐term fermentation. Thus, to mimic a real meal (i.e., porridge), two diets were formulated with lower percentages of non‐fermented (Q‐2) and fermented‐dry roasted quinoa flour (FQ‐2), the other ingredients in both diets were lactose‐free milk powder, corn starch and cane sugar. These diets were also compared with a phytate‐free reference diet (R‐2) based on milk powder (lactose‐free) and corn starch. All the diets were formulated to fulfill growing rats' requirements for protein (50–150 g kg^−1^ diet), energy (15.9–16.7 MJ kg^−1^ diet), zinc (12–25 mg kg^−1^ diet), and iron (35–75 mg kg^−1^ diet) (Council 1995).

**TABLE 1:** Experimental diet formulation (g kg^−1^ diet).

Diet/Component	Non‐fermented flour	Fermented flour	Cane sugar	Sunflower oil	Lactose‐free milk powder	Corn starch
Study 1^a^						
Q‐1	920	—	40	40	—	—
FQ‐1	—	920	40	40	—	—
C‐1	795	—	165	40	—	—
FC‐1	—	795	165	40	—	—
Study 2^b^						
Q‐2	600	—	80	—	170	150
FQ‐2	—	600	80	—	170	150
R‐2	—	—	—	—	500	500

Abbreviations: C‐1, diet prepared with canihua flour dry roasted in a pan at 120°C for 3 min; FC‐1, diet prepared with canihua flour fermented for 12 h and dry roasted in a pan at 120°C for 3 min; FQ‐1, diet prepared with quinoa flour fermented for 24 h and dry roasted in a pan at 120°C for 3 min; FQ‐2, diet prepared with quinoa flour fermented for 4 h followed by dry roasting at 120°C for 3 min; Q‐1, diet prepared with quinoa flour dry roasted in a pan at 120°C for 3 min; Q‐2, diet prepared with quinoa flour dry roasted at 120°C for 3 min; R‐2, reference diet prepared with lactose‐free milk powder and corn starch.

^a^ *n* = 6 animals per group.

^b^ *n* = 8 animals per group.

### 2.2 Animals and in vivo Assay

The animal assay was reviewed and approved by the National Research Ethics Committee in La Paz, Bolivia “Comité National de Bioética, Comisión de Ética de la Investigación, CEI.” The animal studies were conducted in accordance with the “animal research protocols” and “national guidelines for the care and use of laboratory animals.” Wistar rats had an initial body weight of 60 ± 5 g. In study 1, 24 animals were randomly allocated in groups to follow the diets Q‐1, FQ‐1, C‐1, and FC‐1. In study 2, animals were allocated in three groups (*n* = 8): Q‐2, FQ‐2, and R‐2. Individual steel cages (22 × 29 × 20 cm) were used for each animal in the animal care facility. The temperature in the facility was kept at 22°C ± 2°C, and light–dark cycles lasting 12 h. Animals were offered a daily weight feed (daily intake was recorded) and water was given *ad libitum*, the assay was conducted for 30 days.

**FIGURE 1:**
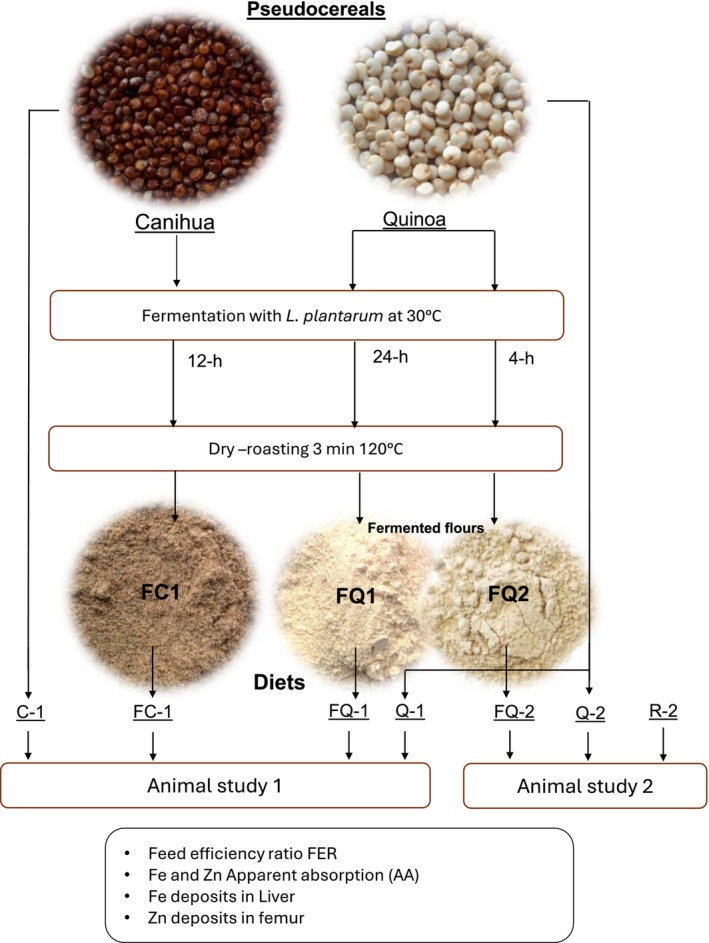
Schematic representation of the diets and animal studies. C‐1, Diet prepared with canihua flour dry roasted in a pan at 120°C for 3 min; FC‐1, diet prepared with canihua flour fermented for 12 h and dry roasted in a pan at 120°C for 3 min; FQ‐1, diet prepared with quinoa flour fermented for 24 h and dry roasted in a pan for at 120°C for 3 min; FQ‐2, diet prepared with quinoa flour fermented for 4 h followed by dry roasting at 120°C for 3 min; Q‐1, diet prepared with quinoa flour dry roasted in a pan at 120°C for 3 min; Q‐2, diet prepared with quinoa flour dry roasted for at 120°C for 3 min; R‐2, reference phytate‐free diet, prepared with lactose‐free milk powder and corn starch.

The in vivo assay to determine the absorption and retention of minerals was adapted from previous studies (McClung et al. 2006; Tesan et al. 2009). Body weight and feed intake were followed and recorded daily to determine body weight gain and feed efficiency ratio (FER). After the 30 days, the animals were euthanized and the liver and the right femur of each animal were collected for iron and zinc analysis. In study 2, feces were daily, collected, weighed, and recorded for each animal. “Apparent absorption” (AA) of iron and zinc was calculated based on the intake and excretion of these minerals. A schematic representation of the diets and study design is shown in Figure 1.

### 2.3 Analytical Procedures

Standardized AOAC methods (Horwitz and Latimer 2000) were used for the analyses of protein, fat and ashes. Carbohydrates were calculated by difference. Energy values were calculated using the following caloric factors for protein 16.7 kJ g^−1^, fat 37.4 kJ g^−1^ and carbohydrates 16.7 kJ g^−1^.

Phytate was determined in the samples of non‐fermented and fermented diets using high‐performance ion chromatography (HPIC) (Carlsson et al. 2001). The phytate content results were used to estimate the iron and zinc bioavailability by calculating the phytate:mineral molar ratios. Phytate:iron (Phy:Fe), phytate:zinc (Phy:Zn) and phytate·calcium:zinc (Phy·Ca:Zn) molar ratios were computed with 660 g mol^−1^ as the molecular weight of phytate. These molar ratios were then compared with the suggested molar ratios of Phy:Fe < 1, Phy:Zn < 15 and Phy·Ca:Zn < 200 for “adequate bioavailability of iron and zinc” (Brown et al. 2004; Hurrell and Egli 2010).

Iron and zinc contents were determined in the diets, liver, femur and feces according to the procedure described by Lazarte, Vargas, and Granfeldt (2015). Briefly, the samples were wet digested with HNO_3_ and H_2_O_2_ in a microwave reaction system “Multiwave PRO” (Anton Paar CO., Ashland, VA, USA). After digestion, the samples were diluted with deionized water. Then iron and zinc were quantified by flame atomic absorption spectrophotometry “AAnalyst 200” (Perkin Elmer Corp., Norwalk, CT, USA) at 248.3 and 213.9 nm wavelength, respectively. Certified atomic absorption standard solutions 1000 ppm (Merck KGaA, Darmstadt, Germany) were used to prepare 5‐point calibration curves in the range 100–2000 mg l^−1^.

### 2.4 Statistical Analysis

The results are presented as mean and standard error of mean. The significant difference of data between non‐fermented and fermented diets for each type of pseudocereal was tested by bivariate analysis *t*‐test. The difference among four diets in study 1 and three diets in study 2 was tested by multivariate analysis using one‐way ANOVA followed by *post hoc* analysis Tukey test (SPSS Inc., IBM Corporation, Armok, USA) to evaluate significant effects. Pearson correlations were computed to elucidate the association between body weight and feed intake, zinc retention in the femur and phytate content and Phy:Zn and Phy·Ca:Zn molar ratios, iron retention in the liver and iron content in the diet. Simple and multiple linear regression analyses were conducted to determine associations between phytate, lactic acid content and mineral content in the diets and mineral content in the liver and femur of the animals. The significance level for all the analysis was set up at *p* values < 0.05 and the SPSS statistical software was used (IBM Corporation).

## 3 Results

Nutrient composition and phytate content of experimental diets used in studies 1 and 2 are shown in Tables 2 and 3, respectively. The main difference in the analyzed nutrients and antinutrients between fermented and non‐fermented diets was the phytate content; that was due to the percentage of either non‐fermented or fermented pseudocereal flour in the diets. It has been proven that the phytate content in pseudocereals significantly decreased during lactic acid fermentation (Castro‐Alba, Lazarte, Perez‐Rea, Carlsson, et al. 2019). This reduction in phytate content improved the estimated bioavailability of minerals, as is shown by the calculated molar ratios. Phy:Zn molar ratios were reduced from above 15.6 (Q‐1) and 17.5 (C‐1) to 3.35 for the fermented quinoa diet (FQ‐1) and 7.64 for the fermented canihua diet (FC‐1). A similar reduction was found for the Phy:Fe molar ratios, indicating an improvement in the estimated bioavailability of these minerals in the diets.

**TABLE 2:** Nutrient and phytate content of non‐fermented and fermented quinoa and canihua diets used to feed the rats during study 1. Mean ± SD expressed in dry matter.

	Q‐1	FQ‐1	C‐1	FC‐1
Energy (MJ kg^−1^)	17.2 ± 0.002	17.5 ± 0.01	16.0 ± 0.001	15.8 ± 0.006
Protein (g kg^−1^)	133 ± 0.10	139 ± 0.20	134 ± 0.10	146 ± 0.20
Fat (g kg^−1^)	76 ± 0.20	89 ± 0.50	115 ± 0.01	107 ± 0.30
Ash (g kg^−1^)	25 ± 0.10	25 ± 0.10	23 ± 0.10	22 ± 0.03
Carbohydrates (g kg^−1^)	765 ± 0.30	747 ± 0.30	728 ± 0.10	724 ± 0.10
Iron (mg kg^−1^)	43.8 ± 0.90	42.8 ± 0.50	103 ± 5.8	104 ± 7.0
Zinc (mg kg^−1^)	44.2 ± 0.30	43.4 ± 0.40	36.4 ± 0.20	35.9 ± 0.70
Calcium (g kg^−1^)	0.60 ± 0.006	0.60 ± 0.01	1.20 ± 0.05	1.24 ± 0.03
Phytate (g kg^−1^)	7.39 ± 0.36^b^	1.47 ± 0.12^a^	6.30 ± 0.18^b^	2.75 ± 0.20^a^
Phy:Zn	15.6 ± 0.80^b^	3.35 ± 0.24^a^	17.5 ± 0.49^b^	7.48 ± 0.55^a^
Phy:Fe	14.3 ± 0.69^b^	2.90 ± 0.24^a^	5.13 ± 0.14^b^	2.26 ± 0.17^a^
Phy:Ca	0.75 ± 0.03^b^	0.15 ± 0.01^a^	0.32 ± 0.01^b^	0.14 ± 0.01^a^
Phy·Ca:Zn	247 ± 14^b^	49.9 ± 3.9^a^	527 ± 15^b^	225 ± 16^a^

*Note:* Different letters in each parameter indicate significant differences at *p* < 0.05 between non‐fermented and fermented quinoa and canihua diets.

Abbreviations: C‐1, diet prepared with canihua flour dry roasted in a pan for at 120°C for 3 min; FC‐1, diet prepared with canihua flour fermented for 12 h and dry roasted in a pan for at 120°C for 3 min; FQ‐1, diet prepared with quinoa flour fermented for 24 h and dry roasted in a pan at 120°C for 3 min; Q‐1, diet prepared with quinoa flour dry roasted in a pan for at 120°C for 3 min.

**TABLE 3:** Nutrient and phytate content of non‐fermented and fermented quinoa and reference diets used to feed the rats during study 2. Mean ± SD expressed in dry matter.

	Q‐2	FQ‐2	R‐2
Energy (MJ kg^−1^)	1.78 ± 0.01	17.9 ± 0.01	18.2 ± 0.01
Protein (g kg^−1^)	128 ± 0.10	129 ± 0.10	133 ± 0.10
Fat (g kg^−1^)	84 ± 0.04	84 ± 0.05	134 ± 0.10
Carbohydrates (g kg^−1^)	742 ± 0.40	746 ± 0.10	620 ± 0.10
Iron (mg kg^−1^)	34.9 ± 1.2	35.4 ± 1.2	9.90 ± 0.40
Zinc (mg kg^−1^)	28.7 ± 0.10	28.6 ± 0.20	20.1 ± 0.30
Calcium (g kg^−1^)	1.07 ± 0.01	1.09 ± 0.03	5.30 ± 0.10
Lactic acid (g kg^−1^)	7.46 ± 0.12	24.1 ± 0.20	0.75 ± 0.05
Phytate (g kg^−1^)	4.75 ± 0.11^b^	1.32 ± 0.03^a^	—
Phy:Zn	27.3 ± 0.73^b^	7.64 ± 0.22^a^	—
Phy:Fe	19.3 ± 0.46^b^	5.29 ± 0.24^a^	—
Phy:Ca	0.45 ± 0.02^b^	0.12 ± 0.004^a^	—
Phy·Ca:Zn	728 ± 38^b^	209 ± 26^a^	—

*Note:* Different letters in each parameter indicate significant differences at *p* < 0.05 between non‐fermented and fermented quinoa and canihua diets.

Abbreviations: FQ‐2, Diet prepared with quinoa flour fermented for 4 h followed by dry roasting at 120°C for 3 min; Q‐2, Diet prepared with quinoa flour dry roasted at 120°C for 3 min; R‐2, Reference diet prepared with lactose‐free milk powder and corn starch.

The results of study 1 (Table 4) showed the effect of feed intake on the body weight gain of four animal groups fed with quinoa (Q‐1 and FQ‐1) or canihua (C‐1 and FC‐1) diets. Rats fed with non‐fermented quinoa and canihua diets consumed bigger amounts of diet each day and were between 36% and 40% heavier than those fed fermented diets, which means that the increase in body weight was positively correlated with feed intake (*r* = 0.715, *p* < 0.01). Mineral retention in the liver and femur of the animals was adjusted by total body weight and mineral intake. Iron concentration in the liver of animals fed diet containing fermented pseudocereals (FC‐1 and FQ‐1) was 43% and 52% higher than in animals fed diet prepared with non‐fermented canihua or quinoa (C‐1 and Q‐1), respectively. Zinc concentration in the femur was similar for non‐fermented and fermented pseudocereal diets. Among all groups, iron concentration in the livers and femurs of animals fed with FQ‐1 diet was the highest.

**TABLE 4:** Effect of non‐fermented and fermented diets on feed efficiency ratio (FER), and iron and zinc retention in femur and liver of Wistar rats in study 1. Mean ± SEM expressed in dry matter.

	Q‐1	FQ‐1	*p* ^d^	C‐1	FC‐1	*p* ^d^
Body weight gain (g)	78.0 ± 3.9^bB^	49.6 ± 3.6^aA^	0.000	111 ± 6.7^bC^	66.7 ± 1.3^aB^	0.000
Feed intake (g)	297 ± 8.7^aA^	294 ± 11^aA^	1.000	414 ± 13^bC^	367 ± 11^aB^	0.018
FER^a^	0.26 ± 0.008^bB^	0.17 ± 0.009^aA^	0.000	0.27 ± 0.009^bB^	0.18 ± 0.005^aA^	0.000
Femur weight (mg g^−1^ BW^b^)	1.35 ± 0.05^aA^	1.46 ± 0.05^aA^	0.172	1.29 ± 0.04^aA^	1.29 ± 0.05^aA^	0.828
Liver weight (mg g^−1^ BW)	12.8 ± 0.49^aA^	15.7 ± 0.55^bB^	0.003	14.6 ± 0.53^aAB^	15.9 ± 0.44^aB^	0.078
Fe liver (μg g^−1^ Fe_Int_ ^c^ g^−1^ BW)	1050 ± 53^aB^	1604 ± 112^bC^	0.001	406 ± 25^aA^	580 ± 43^bA^	0.006
Fe femur (μg g^−1^ Fe_Int_ g^−1^ BW)	10.3 ± 0.80^aB^	20.6 ± 2.7^bC^	0.005	32.0 ± 0.2^aA^	44.0 ± 0.30^bA^	0.015
Zn in liver (μg g^−1^ Zn_Int_ g^−1^ BW)	96.6 ± 4.4^aAB^	129 ± 11^bC^	0.021	93.0 ± 1.7^aA^	123 ± 8.7^bBC^	0.007
Zn femur (μg g^−1^ Zn_Int_ g^−1^ BW)	25.7 ± 2.3^aA^	26.0 ± 2.2^aA^	0.840	20.7 ± 1.0^aA^	21.8 ± 0.84^aA^	0.437

*Note:* Bivariate analysis (*t*‐test) shows differences between non‐fermented and fermented diets, by small letters in each parameter. Multivariate analysis (ANOVA) show by capital letters in each parameter, indicate significant differences between groups at *p* < 0.05.

Abbreviations: C, diet prepared with canihua flour dry roasted in a pan for 3 min 120°C; FC, diet prepared with canihua flour fermented for 12 h and dry roasted in a pan at 120°C for 3 min; FQ‐1, diet prepared with quinoa flour fermented for 24 h and dry roasted in a pan at 120°C for 3 min; Q‐1, diet prepared with quinoa flour dry roasted in a pan at 120°C for 3 min.

^a^ FER, feed efficiency ratio, calculated as body weight gain divided by feed intake.

^b^ BW, body weight.

^c^ Int, adjusted to the intake.

^d^ *p*‐value for the pairwise comparison of non‐fermented and fermented quinoa and canihua.

Study 2 was conducted to investigate the effects of diets containing non‐fermented and fermented quinoa at lower percentages than in Study 1, in a way that diets mimic a real meal, such as quinoa porridge. The additional ingredients in these diets were milk powder (lactose‐free), corn starch and cane sugar. Table 5 shows the effect of feed intake on the body weight gain of two animal groups fed Q‐2 and FQ‐2 quinoa diets with different phytate content, compared with a phytate‐free reference diet (R‐2) based on lactose‐free milk powder and corn starch. Body weight gain was positively correlated (*r* = 0.976, *p* < 0.01) to feed consumption. Feed efficiency ratio of animals fed with Q‐2 and R‐2 diets was similar, and significantly higher than that of the groups fed the FQ‐2 diet. There was no significant difference in the apparent absorption of iron and zinc between animals fed FQ‐2 or Q‐2 diet (Table 5). The concentration of iron and zinc in the liver and zinc concentration in the femur of animals fed with fermented diet (FQ‐2) was significantly (*p* < 0.05) higher than that of non‐fermented diet (Q‐2). The iron content in liver and zinc in femur of animals fed with FQ‐2 diet, which had a low phytate content, was similar to the reference diet (R‐2), which had no phytate in its composition.

**TABLE 5:** Effect of quinoa diets (non‐fermented and fermented) and reference diet on feed efficiency ratio, iron and zinc apparent absorption and mineral retention in liver and femur of Wistar rats in study 2. Mean ± SEM expressed in dry matter.

	Q‐2	FQ‐2	R‐2	*p* ^a^
Body weight gain (g)	93.3 ± 7.0^bB^	53.0 ± 3.9^aA^	109 ± 2.8^B^	0.000
Feed intake (g)	363 ± 15.6^bB^	285 ± 10.2^aA^	388 ± 9.2^B^	0.001
FER^b^	0.26 ± 0.009^bB^	0.18 ± 0.008^aA^	0.28 ± 0.005^B^	0.000
Femur weight (mg g^−1^ BW^c^)	1.37 ± 0.04^bA^	1.61 ± 0.06^aC^	1.57 ± 0.04^B^	0.000
Liver weight (mg g^−1^ BW)	13.8 ± 0.38^aB^	12.8 ± 0.27^aAB^	11.8 ± 0.22^A^	0.082
Fe intake (mg)	11.9 ± 0.51^bC^	9.39 ± 0.34^aB^	3.84 ± 0.09^A^	0.001
Fe excretion (mg)	5.17 ± 0.34^bC^	3.88 ± 0.34^aB^	1.59 ± 0.16^A^	0.001
%FeAA	56.1 ± 2.8^aA^	59.0 ± 2.6^aA^	58.9 ± 3.7^A^	0.464
Fe liver (μg g^−1^ Fe_Int_ ^d^ g^−1^ BW)	1429 ± 150^aA^	2220 ± 224^bB^	2255 ± 183^B^	0.011
Fe femur (μg g^−1^ Fe_Int_ g^−1^ BW)	14.5 ± 1.1^aA^	21.4 ± 1.2^bB^	18.0 ± 1.6^B^	0.001
Zn intake (mg)	9.77 ± 0.42^bB^	7.58 ± 0.27^aA^	7.09 ± 0.16^A^	0.001
Zn excretion (mg)	5.69 ± 0.49^bB^	3.92 ± 0.26^aA^	3.64 ± 0.36^A^	0.001
%ZnAA	44.8 ± 2.4^aA^	48.3 ± 2.6^aA^	44.7 ± 2.1^A^	0.326
Zn in liver (μg g^−1^ Zn_Int_ g^−1^ BW)	89.7 ± 4.6^aA^	103 ± 4.3^aA^	105 ± 4.8^A^	0.054
Zn femur (μg g^−1^ Zn_Int_ g^−1^ BW)	34.5 ± 2.0^aA^	53.2 ± 2.9^bB^	58.2 ± 2.5^B^	0.000

*Note:* Bivariate analysis (*t*‐test) shows differences between non‐fermented and fermented diets, by small letters in each parameter. Multivariate analysis (ANOVA) show by capital letters in each parameter, indicate significant differences between groups at *p* < 0.05.

Abbreviations: FQ‐2, diet prepared with quinoa flour fermented for 4 h followed by dry roasting at 120°C for 3 min; Q‐2, diet prepared with quinoa flour dry roasted at 120°C for 3 min; R‐2, reference diet prepared with lactose‐free milk powder and corn starch.

^a^ *p*‐value for the pairwise comparison of non‐fermented and fermented quinoa.

^b^ FER, feed efficiency ratio, calculated as body weight gain divided by feed intake.

^c^ BW, body weight.

^d^ Int, intake.

To investigate the associations between phytate, lactic acid and mineral content in diets and the iron and zinc accumulation in the liver and femur of the animals, simple and multiple regression analysis was conducted. The independent variables were the phytate and mineral concentration in the diets. Table 6 shows the results of the linear regression; it seems that the retention of iron in the liver was mainly affected by iron content and lactic acid while the retention of zinc in the femur was mainly influenced by the phytate concentration in the diets.

**TABLE 6:** Simple and multiple regression equations for iron and zinc content in the liver and femur of animals fed non‐fermented and fermented diets in study 2.

Dependent factor	Regression equation	*R* ^2^	*p*
**Iron**			
Iron in liver (mg g^−1^ BW)	= 0.013 + 0.001 Phy_Conc_	0.141	0.071
	= 0.010 + 4.51 × 10^−4^ La_Conc_	0.544	0.000
	= 0.004 + 0.437 Fe_Conc_	0.609	0.000
	= 0.006 + 2.26 × 10^−4^ La_Conc_ + 0.281 Fe_Conc_	0.664	0.058, 0.012
Iron in femur (mg g^−1^ BW)	= 1.15 × 10^−4^ + 1.494 × 10^−5^ Phy_Conc_	0.254	0.012
	= 1.887 × 10^−5^+ 0.005 Fe_Conc_	0.828	0.000
	= 7.956 × 10^−6^—8.51 × 10^−6^ Phy_Conc_ + 0.006 Fe_Conc_	0.877	0.015, 0.000
**Zinc**			
Zinc in liver (mg g^−1^ BW)	= 0.001 + 2.691 × 10^−5^ Phy_Conc_	0.365	0.002
	= 0.001 + 0.010 Zn_Conc_	0.179	0.033
	= 0.001 + 2.755 × 10^−5^ Phy_Conc_—4.38 × 10^−4^ Zn_Conc_	0.366	0.021, 0.940
Zinc in femur (mg g^−1^ BW)	= 4.14 × 10^−4^—1.686 × 10^−5^ Phy_Conc_	0.665	0.000
	= 4.76 × 10^−4^ + 3.61 × 10^−7^ La_Conc_	0.007	0.690
	= 0.001–0.005 Zn_Conc_	0.275	0.008
	= 3.78 × 10^−4^—1.935 × 10^−5^ Phy_Conc_ + 0.0017 Zn_Conc_	0.677	0.000, 0.380

Abbreviations: BW, body weight; Fe_Conc_, iron concentration in the diet, mg g^−1^ diet; La_Conc_, lactic acid concentration in the diet, mg g^−1^ diet; Phy_Conc_, phytate concentration in diet, mg g^−1^ diet; Zn_Conc_, zinc concentration in the diet, mg^−1^g diet.

## 4 Discussion

The main findings of the research indicate that the diet based on roasted fermented quinoa (FQ‐2) performed as good as the reference diet (R‐2), which was phytate‐free. Both diets showed similar indicators of mineral bioavailability vis. retention of iron in the liver and femur and retention of zinc in the femur. These diets showed higher iron and zinc retention than those obtained from the diet based on non‐fermented quinoa (Q‐2). The results in study 1 also showed that the retention of iron was higher in the liver and femur of animals fed the fermented quinoa diet, followed by the group fed non‐fermented quinoa. The groups that consumed fermented quinoa or fermented canihua showed higher retention of zinc in the liver than the groups that consumed non‐fermented diets. Furthermore, linear regression analysis (study 2) indicated that iron accumulation in the liver and femur was affected by iron content and to a lesser degree by lactic acid concentration in the diets. While zinc accumulation in the femur was negatively associated with the phytate content of the diets. Higher zinc retention in the femur was found in rats after fermented quinoa diet (FQ‐2), with reduced phytate content, and not significantly different to the results obtained after the phytate‐free reference diet. Thus, the results showed that the inclusion of fermented quinoa or canihua improved the bioavailability of iron and zinc in growing rats.

The in vivo assay also provided information on the influence of the addition of fermented food on body weight gain (BWG) and feed intake (FI). It was observed (Tables 4 and 5) that the rats fed fermented diets had significantly lower BWG (*p* < 0.05) than the animals fed non‐fermented diets. It should also be mentioned that the intake of fermented diet was significantly lower (*p* < 0.05) than the non‐fermented diet. Feed efficiency ratio (FER) was influenced by fermentation but not by the type of pseudocereal. Animals in the group of diets prepared with fermented flours exhibited inferior FER than after non‐fermented diet. The low feed intake, in this study, might be related to the increased satiety due to consumption of fermented pseudocereals, quinoa, or canihua. It has been shown that fermented pseudocereals had a higher content of lactic acid (Ayub, Castro‐Alba, and Lazarte 2021; Castro‐Alba, Lazarte, Perez‐Rea, Carlsson, et al. 2019) and likely other organic acids. Carciochi et al. (2016) have reported that fermentation of quinoa raised the total content of phenolic compounds and antioxidant capacity. A recent study showed that fermentation of white quinoa significantly increased total phenolic content from 4.68 to 7.78 mg GAE g^−1^, while the antioxidant capacity increased from 4.83 to 6.91 mg TE g^−1^ (Chu et al. 2024). It was stated that phenolic compounds can be transformed from bound form to a free state, this change has been attributed to bond breakdown, microbial metabolism and enzymatic activity during fermentation (Melini and Melini 2021). A recent systemic review suggests that fermented foods may regulate appetite, mainly due to the lactic and acetic acids produced during fermentation (Chatonidi, Poppe, and Verbeke 2023). It has been discussed that intake of fermented diet decreased gastric pH, a low gastric pH may, in turn, reduce the rate of gastric emptying and magnify the activity or proteases in the stomach (Missotten et al. 2015). In a study, in which the effect of fermented barley on the reduction of body weight in rats was investigated (Zhang et al. 2016), the diet containing fermented barley showed a lower increment in body weight in rodents by reducing body fat. The authors attributed the effect to the higher levels of phenolic compounds found in fermented barley. Other authors have found that phenolic compounds in cereal have a suppressive effect on body weight and obesity (Vitaglione, Napolitano, and Fogliano 2008). Furthermore, in a recent published study, it was shown that fermentation of quinoa increased dietary fiber content from 9.11% to 15.48% (Maldonado‐Alvarado et al. 2023). Higher dietary fiber is reported to contribute to an increased satiety, various endocrine and mechanical signals from the gastrointestinal tract are aroused by fibers and by their fermentation products, which then reach sites of brain to reduce food intake (Akhlaghi 2024).

In our previous studies (Ayub, Castro‐Alba, and Lazarte 2021; Castro‐Alba, Lazarte, Perez‐Rea, Carlsson, et al. 2019; Castro‐Alba, Lazarte, Perez‐Rea, Sandberg, et al. 2019), it has been shown that lactic acid fermentation had a positive effect on reducing the phytate content of pseudocereals. This reduction was mainly due to endogenous phytase, which was activated as a result of the reduction of pH during fermentation of pseudocereals with *L*. *plantarum*. Activated phytase has the ability to hydrolyze phytate to less phosphorylated compounds, which are more soluble at intestinal pH, and release minerals. During fermentation, organic acids are also produced and some of them are reported to have a positive effect foremost on the bioavailability of iron (Teucher and Cori 2004) but under certain conditions also on zinc (Affonfere et al. 2023).

Mineral accumulation in the liver and femur has been previously used as an indicator to evaluate the bioavailability of iron and zinc in rats (Ammerman, Baker, and Lewis 1995). In study 1 of the present investigation, the fermented diets FQ‐1 and FC‐1 contained 92% and 79.5% of fermented quinoa or fermented canihua, respectively. The results showed that iron retention in the liver and femur of animals was significantly higher in the groups after being fed the diet containing fermented quinoa, but not after being fed the diet containing fermented canihua. The diets in study 2, in which fermented quinoa was added at 60%, performed better in retention of iron than the diets in study 1. Iron concentration in the liver was correlated, positively, with iron content in the diet (*r* = 0.780, *p* < 0.01). Furthermore, linear regression analysis (Table 6) suggests that iron concentration in the liver had a positive association with iron content in the diet (*β* = 0.437 mg g^−1^, *p* = 0.000), and lactic acid content in the diet (*β* = 4.51 × 10^−4^ mg g^−1^, *p* = 0.000). However, no significant association was found between iron retention in liver and phytate content in the diet. This aligns with the work of other authors (Hurrell et al. 1992), which showed that phytate content must be virtually zero to significantly improve iron absorption. It has been emphasized that for iron absorption to improve significantly, the phytate content in food must be reduced to almost zero because phytate strongly inhibits the absorption of iron by forming insoluble complexes with it (Hurrell 2004).

The significant variables of iron and lactic acid content in the diet were computed in a multiple regression analysis. The results show that the most significant variable, to modulate iron bioavailability, was iron content (*p* = 0.000), followed by lactic acid content in the diet (*p* = 0.058). It has previously reported that iron concentration in liver of rats is related to the absorption of dietary iron, which is associated with the solubility of iron in the gastrointestinal tract (Schlemmer et al. 2009). It has also been suggested that iron absorption and retention, independent of phytate reduction, can be improved by organic acids or changes in iron speciation as a result of fermentation (Affonfere et al. 2023; Scheers et al. 2016). Accordingly, the diet made of fermented quinoa (24.1 g kg^−1^) had a higher content of lactic acid than the non‐fermented diet (7.46 g kg^−1^) and probably also of other organic acids. In a previous study (Castro‐Alba, Lazarte, Perez‐Rea, Carlsson, et al. 2019), we have shown that lactic acid fermentation of pseudocereals is a successful processing technique for decreasing phytate content and, therefore, enhancing iron accessibility. We reported that the in vitro accessibility of iron in fermented quinoa was 3.6‐fold higher than that of non‐fermented quinoa (Castro‐Alba, Lazarte, Perez‐Rea, Carlsson, et al. 2019). As indicated, the rationale behind the results was that fermentation of raw pseudocereal activated its endogenous phytase, which then hydrolyzed phytate to smaller inositol phosphates leading to the release of minerals in free form. Moreover, the formation of lactic acid and other organic acids during fermentation have a positive effect on accessibility, absorption and bioavailability of iron, because lactic acid can form soluble ligands with iron (Hemalatha, Platel, and Srinivasan 2007; Hussain et al. 2024). Further, in study 2, iron retention in the liver of animals fed the FQ‐2 diet was comparable to that of animals fed the reference diet R‐2, and significantly higher than in non‐fermented quinoa diet. Also in study 2, where fermented quinoa was added at 60%, performed better in retention of zinc than the diets in study 1, where the percentage of fermented pseudocereals was as high as 92%. This could be due to the zinc concentration in the quinoa and canihua diets (36–43 mg kg^−1^) was higher than the recommended value (12–25 mg kg^−1^) for growing rats. It is indicated that excess iron or zinc in the diets can reduce their bioavailability (Affonfere et al. 2023).

The results from study 2 showed that animals fed a FQ‐2 diet had a significantly higher zinc content in the femur than animals fed a Q‐2 diet. Moreover, zinc accumulation in the femur was negatively associated with phytate content (*r* = −0.815, *p* < 0.01), the molar ratios followed a similar trend Phy:Zn (*r* = −0.815, *p* < 0.01) and Phy·Ca:Zn (*r* = −0.814, *p* < 0.01). The simple linear regression analysis shown in Table 6 suggests that zinc content in the femur exhibited an inverse correlation with phytate in the diet (*β* = −1.686 × 10^−5^, *p* = 0.000) as well as with zinc content in the diet (*β* = −0.005, *p* = 0.008). In addition, when both independent variables (phytate and zinc content in the diet) were computed in a multiple regression analysis, the main effect was attribuited to phytate content, and zinc content in the diet became not significant. The Q‐2 diet with higher phytate concentration (4.75 g kg^−1^) had in turn higher values of Phy:Zn molar ratio (27.3), thus, the zinc bioavailability was affected by phytate concentration in this diet, resulting in lower zinc accumulation in the femur of the animals. The FQ‐2 diet had a lower phytate concentration (1.32 g kg^−1^) and Phy:Zn molar ratio (7.64) in its composition due to the fact that during fermentation, phytate was hydrolyzed mainly by endogenous phytase activity. The findings in this paper are in line with the result presented by Lazarte, Vargas, and Granfeldt (2015), where an increase in zinc absorption was reported when rodents were fed a fermented cassava diet with a molar ratio Phy:Zn 2.76. Those findings were attributed to the higher zinc bioavailability achieved through phytate hydrolysis, where complex phytate molecules are transformed into smaller phytate forms (IP1, IP2) and zinc is found in its free form (Lazarte, Vargas, and Granfeldt 2015). Other authors (McClung et al. 2006) have also reported a higher zinc content in the femur of animals fed a low‐zinc diet with added phytase, when compared to a diet without added phytase. It was argued that the added phytase hydrolyzed the initial phytate and thus, absorption of zinc was increased. In addition, our results indicate that the diet containing fermented quinoa was comparable to the reference diet (phytate‐free reference) in terms of zinc accumulation in the femur.

## 5 Conclusion

The inclusion of 60% fermented quinoa in the milk diets showed a higher retention of iron in the liver and femur of animals fed a FQ‐2 diet than in those fed Q‐2 diet. This bigger accumulation of iron in the femur was equivalent to the iron retention in femur of animals fed a R‐2, reference diet with no phytates in its content. Regression analysis for iron retention showed that the main factor affecting retention and bioavailability of this mineral was iron concentration in the diet and likely the presence of organic acids in the diet. Accumulation of zinc in the femur of rats was also higher after consuming the FQ‐2 diet and to the same extend after the R‐2 diet, both diets exhibited higher results than those found after the non‐fermented Q‐2 diet. Regression analysis showed that the retention and bioavailability of zinc in rats was mainly dictated by the phytate concentration in the diets. It is also likely that the consumption of diets with fermented pseudocereals increased satiety in rats, this was showed by the lower gain in weight and FER in rats fed fermented pseudocereal diets. However, the lower consumptions of feed and inferior weight gain did not negatively affect the accumulation of minerals in the liver and femur of animals. Thus, the findings indicate that the inclusion of fermented pseudocereals in diets may increase the bioavailability of zinc and iron in Wistar rats. The implications for iron and zinc absorption from fermented pseudocereals in humans need further investigation.

## Author Contributions

**Vanesa Castro‐Alba:** conceptualization (equal), data curation (equal), formal analysis (equal), investigation (equal), methodology (equal), software (equal), writing – original draft (equal), writing – review and editing (equal). **Mirian Vargas:** investigation (equal), methodology (equal), supervision (equal), writing – review and editing (equal). **Ann‐Sofie Sandberg:** resources (supporting), writing – review and editing (supporting). **Daysi Perez‐Rea:** writing – review and editing (supporting). **Björn Bergenståhl:** data curation (supporting), funding acquisition (lead), project administration (lead), supervision (supporting), validation (equal), writing – review and editing (supporting). **Yvonne Granfeldt:** conceptualization (equal), formal analysis (equal), project administration (supporting), supervision (equal), validation (equal), writing – review and editing (equal). **Claudia E. Lazarte:** conceptualization (equal), formal analysis (equal), investigation (equal), methodology (equal), supervision (equal), writing – review and editing (equal).

## Conflicts of Interest

The authors declare no conflicts of interest.

## Data Availability

All data that support the findings of this study are available upon reasonable request to the corresponding author.
